# Exosomes: new targets for understanding axon guidance in the developing central nervous system

**DOI:** 10.3389/fcell.2024.1510862

**Published:** 2025-01-09

**Authors:** Mingyu Liu, Teng Teng

**Affiliations:** ^1^ Key Laboratory of Tropical Translational Medicine and Ministry of Education, Hainan Academy of Medical Sciences, Hainan Medical University, Haikou, China; ^2^ School of Stomatology, Hainan Academy of Medical Sciences, Hainan Medical University, Haikou, China; ^3^ School of Basic Medicine and Life Science, Hainan Academy of Medical Sciences, Hainan Medical University, Haikou, China; ^4^ Department of Histology and Embryology, Hainan Academy of Medical Sciences, Hainan Medical University, Haikou, China

**Keywords:** axon guidance, central nervous system development, exosomes, neurodevelopmental disorders, neuronal circuit formation

## Abstract

Axon guidance is a key event in neural circuit development that drives the correct targeting of axons to their targets through long distances and unique patterns. Exosomes, extracellular vesicles that are smaller than 100 nm, are secreted by most cell types in the brain. Regulation of cell-cell communication, neuroregeneration, and synapse formation by exosomes have been extensively studied. However, the interaction between exosomes and axon guidance molecules is poorly understood. This review summarizes the relationship between exosomes and canonical and non-canonical guidance cues and hypothesizes a possible model for exosomes mediating axon guidance between cells. The roles of exosomes in axon outgrowth, regeneration, and neurodevelopmental disorders are also reviewed, to discuss exosome-guidance interactions as potential clinical therapeutic targets.

## 1 Introduction

The complexity and correct connectivity of neural circuits ensures that the brain operates correctly. During neural circuit development, differentiating neurons extend their axons to encounter their appropriate targets to form a functional synapse, which is the basis of neural function in both health and disease (Eisenbach et al., 2004; Kandel, 2005). The faulty assembly of synapses or misdirection of axonal pathfinding leads to defects in neural circuits that may result in neural disorders, including schizophrenia and autism spectrum disorder (ASD) (Gliman et al., 2012; Bakos et al., 2015). In the last decade, genome-wide association studies (GWAS) on developmental and degenerative neural disorders have identified candidate risk genes in the developing neural circuit, highlighting the importance of axonal pathfinding in nervous system development (Bossers et al., 2009; Gliman et al., 2012; Antonell et al., 2013; Pinto et al., 2014; Ronemus et al., 2014; Kashyap et al., 2019). Thus, appropriate axon guidance establishes a heathy and well-functioning neural circuit.

The axon guidance process includes axon genesis, outgrowth, pathfinding, and regeneration. During brain development, billions of axons are guided toward their proper targets by axon guidance molecules that are subdivided into attractive and repulsive molecules. Long-range axon guidance cues are required by axons to migrate throughout the brain, whereas short-range effects are mediated by cell-cell contact-dependent ligand-receptor binding models. Long-range guidance cues are recognized at various intermediate choice points that express guidance molecules. These choice points exert either attractant or repellent effects on axons to regulate axon projection (Squarzoni et al., 2015). Short-range cues function at the growth cones in a region enriched with filopodia (Landis, 1983). Protrusion or collapse of the filopodia controls the forward movement or stopping of the axon, corresponding to attractive or repulsive guidance cues, respectively (Robles, 2005; Suter, 2011; Omotade et al., 2017). The combined long- and short-range effects lead to proper neural circuit formation.

Ramón y Cajal first described axon innervation in the brain and growth cones as the structures that guide billons of axons to their targets (Landis, 1983). What are the factors that regulate all these axons to the correct targets? This question arose a hundred years ago and has attracted the interest of numerous neuroscientists, moving from an understanding on a morphological to molecular basis. Since the 1960s, as the emphasis on guidance cues grew, numerous studies have focused on defining and identifying axon guidance molecules (Katz and Lasek, 1979). In later decades, several axon guidance molecules were discovered and well-studied, including netrin (Harris et al., 1996), ephrin (Zhu et al., 2006), semaphorin (Kolodkin et al., 1993), slit (Wu et al., 1999), and non-conventional cues, such as bone morphogenetic protein (BMP), sonic hedgehog (Shh), and wingless/int-1 (Wnt) (Yam and Charron, 2013). Recently, researchers have identified new guidance molecules, such as draxin, which was determined to provide a repulsive signal for axons in the developing brain and does not share homology with other axon guidance molecules (Shinmyo et al., 2015). Moreover, crosstalk occurs between guidance cues and significant cooperation occurs amongst molecules, such as between netrin and Shh, and netrin and ephrin (Ricolo et al., 2015; Sloan et al., 2015). Updated studies indicate that axon guidance cues also regulate axon projection by mediating changes in gene expression via RNA-binding proteins, which has been well reviewed by Kim and Kim (2020). In addition to being a fundamental feature of neural circuit development, axon guidance contributes to the nerve regeneration process. Nerve injury occurs frequently and often combines with other injury or diseases, and its recovery requires proper axon projection to the original target. Otherwise, the incorrect navigation of an axon leads to misdirection which may result in disrupted recovery (Kerschensteiner et al., 2005; de Ruiter et al., 2008; Hamilton et al., 2011). These studies have established a overview of axon guidance; however, our understanding remains incomplete. Challenges in understanding how a long-range cue precisely guides a single axon to its proper target both spatially and temporally still exist. Moreover, questions remain about how deliberate management is established among numerous intermediate choice points and in signaling crosstalk. To achieve the goals of axon guidance, a missing factor must be present in large quantities with ubiquitous distribution in the brain and contain complex signaling information, which require extracellular transport rather than being ‘fixed’ to tissues or cells and contain abundant essential molecules. On the other hand, when considering clinical treatment, the ability to engineer, isolate, and ensure re-uptake is essential. Exosomes, which are small extracellular vesicles (EVs), are a good candidate that fulfill all these criteria.

Exosomes are a subtype of small EVs with nanoscale sizes ranging between 30 and 100 nm. After their fusion with the plasma membrane, the multivesicular bodies (MVBs) deliver exosomes into the extracellular surroundings, and may undergo reuptake by receptors located on the cell membrane of the recipient (Denzer et al., 2000; Yuan et al., 2018). Exosomes can be released by different cell types, including neurons, stem cells, tumor cells, and immune cells, as well as body fluids, including cerebrospinal fluid (CSF), blood, and urine (Parolini et al., 2009; Mathivanan et al., 2010). With their unique lipid bilayer structure, exosomes are able to carry and protect cargos of biosignaling molecules, including proteins, lipids, and nuclei acids, to deliver information between cells to establish various biological signals (Xia et al., 2019; Gao et al., 2020). Exosomal activity initiates from their fusion with the plasma membrane of recipient cells or by undergoing endocytosis (Xia et al., 2022). This functions as a cell-cell communication feature that provides a contact-independent method to establish signaling between cells (Théry, 2011; Bang and Thum, 2012). Furthermore, as exosomes are detected in body fluids, potential clinical applications may use exosomes as biomarkers for pathological conditions, such as to identify mRNAs in exosomes that are isolated from the serum of patients with glioma (Skog et al., 2008).

During brain development, most types of cells secrete exosomes, including neural stem cells, neurons, astrocytes, and all other types of glia (Kang et al., 2008; Yuyama et al., 2012). Exosomes are involved in regulating cell-cell communication, cell morphology, and plasticity. An increasing number of studies are focusing on the role of exosomes in neural functions, and a study indicates a significant impact of exosomes in neurotransmission (Xia et al., 2019). This study discusses how neural and glial exosomes regulate synapse activity to control neurotransmitter release and highlights the potential function of exosome neurite growth. Brain cancers, including glioblastoma, involve interactions between different cell types in the brain, such as neuron-astrocyte or neuron-microglia interactions. Therefore, exosomes as long-range cell-cell interaction modulators have been linked with brain cancer progression (Kucharzewska et al., 2013). Conversely, exosomes may serve as biomarkers for cancer therapeutics. Moreover, the role of exosomes in Alzheimer’s and Parkinson’s diseases has been well studied, demonstrating the essential contribution of exosomes in neurodegenerative disorders. Overall, as research on exosomes continues to gain popularity, their functions are being further explored, with an increasing focus on clinical therapies (Khan et al., 2022; Zakeri et al., 2024). I believe there is a promising synergy between clinical research and basic science. However, our understanding of the role of exosomes in neural circuit development, particularly in axon guidance, remains limited. Here, we will review current progress that has been made in neuronal exosome research, with a focus on axon guidance and regeneration, summarize the interactions between exosomes and guidance cues, and highlight related neural disorders to discuss the contribution of exosomes in brain development.

## 2 Exosomes and axon guidance cues

**FIGURE 1:**
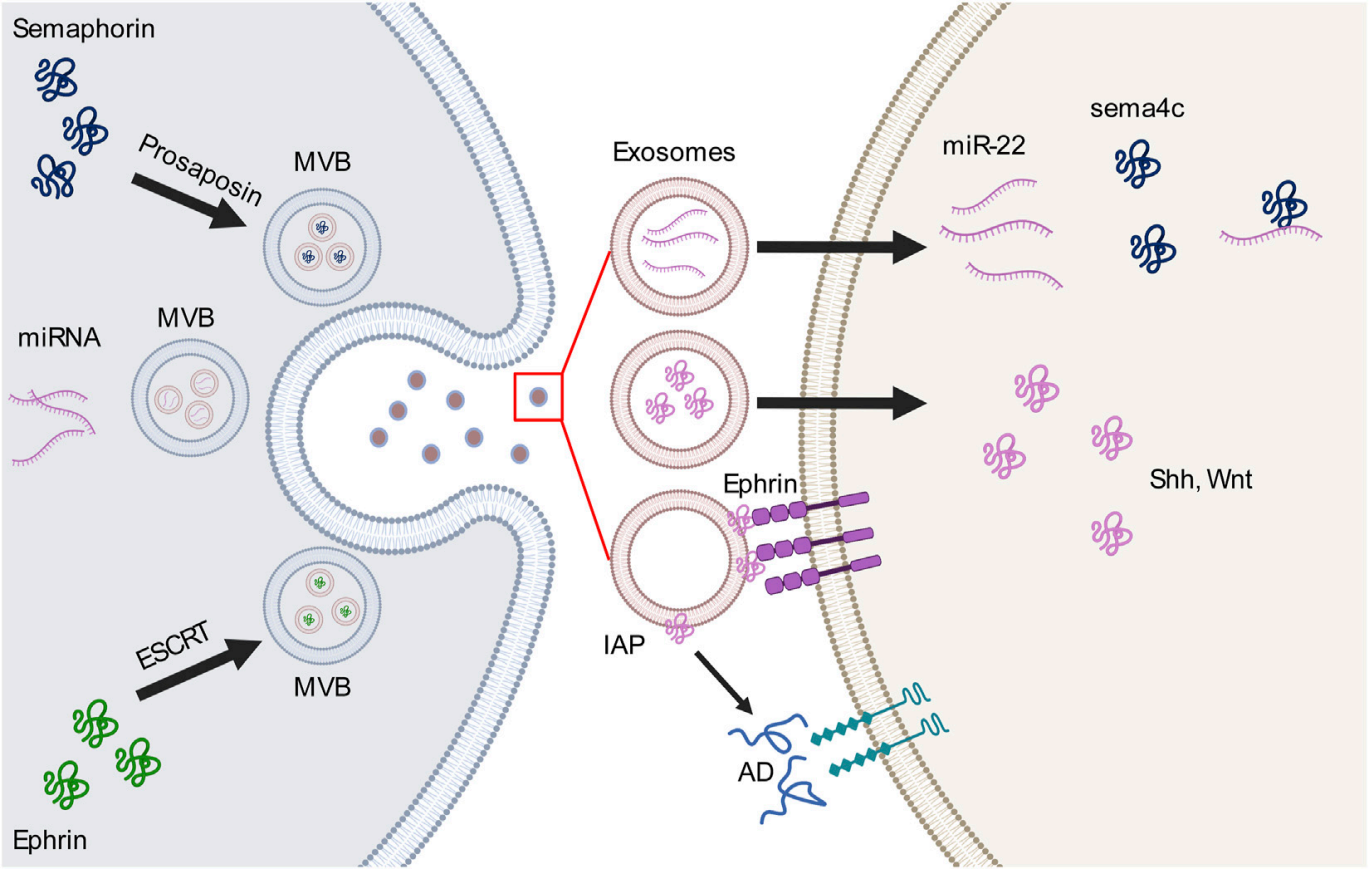
Interactions between exosomes and guidance molecules in the axon guidance pathway. Guidance molecules perticipate in the formation of multivesicular body (MVB) and exosome loading. For example, semaphorin 4A (sema4c) binds to prosaposin to mediate the MVB sorting pathway, whereas EphB2 interacts with the endosomal sorting complex required for transport (ESCRT) which is necessary for MVB formation. MVBs release different types of cargo-loaded exosomes, including microRNAs (miRNAs) which bind to or activate guidance molecules or ligands within the exosome or on the outer surface of the exosomes. These cargos then bind to receptors on the cell membrane or participate in other extracellular pathways.AD, adenosine; IAP, intestinal alkaline phosphatase; Shh, sonic hedgehog; Wnt, wingless-related integration site.

Exosomes have been described to participate in neural circuit development in two ways: as vehicles that transfer molecules (receptors or ligands) or carry cargos of miRNAs to mediate the expression of relevant molecules or activate pathways. The miRNAs carried by various sources of exosomes that participate in axon guidance are summarized in Table 1. In this section, we review the most recent reports of exosomes and guidance molecules in axon guidance (Figure 1).

**TABLE 1:** List of exosomal miRNAs that participate in the axon guidance pathway.

miRNA	Model	Disorder	References
miR-181a, miR-29a	Human	Acute encephalitis	Goswami et al. (2017)
miR-3613-5p, miR-4668-5p, miR-8071, miR-197-5p, miR-4322, and miR-6781-5p	Human	Mesial temporal lobe epilepsy with hippocampal sclerosis	Yan et al. (2017)
miRNA-22a	Zebrafish		Sheng et al. (2022)
miRNA-4262, miR-144-3p, miR-302d-3p, miR-485-5p	Human	Brain metastasis affected by radiotherapy	Li et al. (2021)
miR-19b, miR-148a, miR-150, miR-221, miR-223 miR-320a, miR-361, and miR-486	Cattle		Muroya et al. (2015)
miR210	Human	Breast cancer	Jung et al. (2017)

### 2.1 Exosomes and ephrins

Researches of exosomal Eph-ephrin signaling was initiated a decade ago by Choi et al. (2007), who reported the proteomic profile of a cancer cell line that identified the presence of EphA2-8, EphB1-4, ephrin-B1, and ephrin-B2 on exosomal membranes. Next, Sun et al. (2016) added ephrin-A2 to the story. In the pathogenesis of various cancers, tumor cells release exosomes carrying biological components into the environment to mediate Eph receptor or Ephrin expression, establishing their physiological functions, including cell boundary formation, migration, and axon guidance. For instance, exosomes derived from hypoxic breast cancer cells transport miRNA-210 to neighbor cells, which regulate ephrin-A3 expression to promote angiogenesis (Jung et al., 2017). This exosome-ephrin interaction mechanism has also been confirmed by a study of ephrin-B2 function in endothelial cells (Tae et al., 2017). The most well-studied interaction is exosomal EphA2 derived from tumor serum that contributes to vesicular pathfinding, angiogenesis, and cell migration via Eph-ephrin forward signaling and is a potential diagnostic biomarker for several cancers (Fan et al., 2018; Gao et al., 2021; Han et al., 2022; Wei et al., 2020). In the nervous system, as the largest tyrosine kinase receptor family, Eph-ephrin signaling is implicated in many cellular events during neural circuit development, such as axon guidance and neuron migration via contact-dependent bidirectional signaling. Gong et al. illustrated that exosomes mediate contact-independent Eph-ephrin signaling via a long-range intracellular communication mechanism (Gong et al., 2016). The endosomal sorting complex required for transport (ESCRT) plays an essential role in MVB formation and interacts with EphB2. These EphB2+ exosomes induce ephrin-B1–EphB2 reverse signaling and provide repellent signaling to ephrin-B1+ growth cones. These findings underscore the contribution of exosomes in Eph-ephrin signaling.

### 2.2 Exosomes and semaphorin

A descriptive proteomic analysis of glioma-associated stem cell-derived exosomes identified semaphorin 7A on exosomal membranes. Exosomal semaphorin 7A interacts with integrin-β to increase glioma stem cell (GSC) motility, demonstrating an important role of exosomal semaphorin in neural stem cell migration. This may be a new target for disrupting the interaction between GSCs and neighbor cells (Manini et al., 2019). In addition to their contribution to neuronal migration, exosomal semaphorins regulate vascular and neural pathfinding as guidance cues. Endothelial-derived exosomes carry miRNA-22 as cargo, and the aberrant expression of miRNA-22 leads to disruptions in vascular and motor neuron pathfinding (Sheng et al., 2022). Interestingly, miRNA-22 binds to the 3′-untranslated region of the semaphorin 4C (*sema4c*) gene, highlighting its role in regulating vascular and neural pathfinding through an exosomal pathway (Sheng et al., 2022). Semaphorins also mediate cargo loading of endodermal exosomes. Under oxidative stress, semaphorin 4A transfers prosaposin from the Golgi apparatus to the cell periphery to be loaded and released by exosomes, and Rab11 mediates this intracellular process in retinal pigment epithelial cells. These findings underlines the role of semaphorins in mediating endosomal sorting towards the exosomal pathway (Toyofuku et al., 2012).

### 2.3 Exosomes, netrin, and slit

Netrin-1 was the first axon guidance molecule discovered in vertebrates, making it a significant factor in both central and peripheral nervous system research due to its role in axon guidance, cell migration, and morphogenesis (Serafini et al., 1994). In the peripheral nervous system (PNS), Netrin-1 plays a key role in the upregulation of the nerve stump following peripheral nerve injury (PNI). A recent study demonstrated that exosomes derived from Netrin-1-high endothelial cells (NTN1 EC-EXO) are involved in the formation of the vascular niche. Multi-omics analysis confirmed a low expression of let-7a-5p in NTN1 EC-EXO, highlighting its crucial role in establishing a microenvironment for nerve repair after PNI by activating key signaling pathways such as focal adhesion and axon guidance (Huang et al., 2024). On another hand, Exosomal netrin-1 increases the neuronal differentiation rate of bone marrow mesenchymal stem cells (BMSCs) via Hand2/Phox2b signaling. Moreover, BMSC transplantation potentially repairs the structure of damaged tissue and restores function, which may be used as a supplementary treatment in spinal cord injury or congenital spinal disorders, including spinal bifida aperta (SBA) (Ma et al., 2022). Additionally, more recent research showed that using engineered exosomes enriched with Netrin-1 in SCI rats promoted nerve recovery (Lu et al., 2023). Both results highlight the therapeutic effect of exosomal netrin in neural disorders. Roundabout homolog 1 (ROBO1) acts as the direct target of human engineered exosomal miR-29a-3p, and the interaction between miR-29a-3p and ROBO1 plays an important role in glioma migration and vasculogenic mimicry formation (Zhang et al., 2021). Both Netrin and Slit signaling are classic axon guidance modulators, similar to the ephrin family; however, there is limited research on exosomal Netrin or Slit. As mentioned, Huang’s study highlighted the key role of exosomal Netrin-1 in axon guidance within the vascular niche, underscoring a new direction for research on exosomal axon guidance signaling in the microenvironment of the PNS.

### 2.4 Exosomes and alkaline phosphatase

Alkaline phosphatase (AP), or tissue non-specific alkaline phosphatase (TNAP) is well known as its role in bone development and functions. Transgenic mice lacking TNAP activity display the characteristic skeletal and dental phenotype of infantile hypophosphatasia (Narisawa et al., 1997). In central nerve system, TNAP initially highly expressed in migrating primordial germ cells in neural tube (Foster et al., 2012) and had been demonstrated that played an important role in cell proliferation (Altman and Bayer, 1990), embryonic and adult neurogenesis (Langer et al., 2007), neuronal differentiation (da Silva and Dotti, 2002) and synaptogenesis (Fonta et al., 2005). The role of alkaline phosphatase (AP) in axon guidance has been poorly studied, however, Elefant et al. (2016) demonstrated the attractive role of exosomal AP in the developing avian optic chiasm. During early development of the chick embryo, intestinal alkaline phosphatase (IAP) is localized on the outer surface of exosomes that are highly concentrated in the midline of the developing diencephalon. In a following study by the same group, the attractive role of IAP was confirmed using an *in vitro* culture experiment showing that IAP acts on ambient extracellular adenosine triphosphate (ATP) to form an adenosine gradient from the breakdown of extracellular ATP.

### 2.5 Exosomes and Shh

Shh is a member of the hedgehog (Hh) family and acts as a key morphogen involved in many long-distance cellular events during development, including various cell behaviors and neural plasticity. In various systems, Hh is released and distinctly localized in tissues to activate target genes (Briscoe and Thérond, 2013). Therefore, the transport and release of Hh requires strict spatial and temporal control. In mammals, the Shh morphogen also directs tissue and axonal pattering according to a concentration gradient. This requires the transportation of Shh from producer to recipient cells, and exosomes are the Shh carriers in this extracellular process (Gradilla et al., 2014). Moreover, BMSC-derived exosomal Shh plays a key role in spinal cord injury (SCI) in rats (Jia et al., 2021). Compared with that in the control group, Shh knockdown by short hairpin RNA Shh-adeno-associated virus results in a significantly decreased neuroprotective effect, including a reduced amount of Nissl bodies, lower motor function (according to blood–brain barrier analysis), increased neural apoptosis, and decreased neuronal ends regeneration. This suggests a neuroprotective role of exosomal Shh. However, after regeneration of neuronal ends, axons may contact an inappropriate target, such as misdirection of motor neuron projections after SCI recovery in the case of no exercise or a inefficitent electrical stimulation (Gordon and English, 2016). The next steps in neural injury recovery require further investigation to understand neuronal pathfinding back to their original targets.

### 2.6 Exosomes and Wnt

Wnt is a large family of ligands that play an important role in development, particularly in neuronal development. As a guidance cue, non-canonical Wnt signaling directs several types of axons, including neurons that express dopamine and 5-HT in the hindbrain and corticospinal tract (CST) and post-crossing commissural axons, via gradient expression of different Wnts (Lyuksyutova et al., 2003; Liu et al., 2005; Keeble et al., 2006; Fenstermaker et al., 2010). Non-canonical Wnt signaling (β-catenin-independent) includes Wnt/planar cell polarity (PCP) and Wnt/calcium (Ca^2+^) pathways. The Wnt/PCP pathway is one of the most popular Wnt non-canonical catenin-independent pathways involved in the axon guidance mechanism. In PCP signaling, frizzled (Fz) activates a cascade involving the small GTPases, Rac1 and RhoA, and c-Jun N-terminal kinase to regulate cell polarity and tissue morphogenesis (Yam and Charron, 2013). Dopaminergic and serotonergic axons project from the hindbrain to their midbrain and forebrain targets through a Wnt/PCP pathway (Fenstermaker et al., 2010; Shafer et al., 2011). Gradient-expressed Wnt5a and Wnt7b regulate dopaminergic axon projection as repulsive and attractive cues, respectively; however, axons are not affected in mutants of their receptor, *Fz3* ^*−/−*^. In the Ca^2+^ pathway, Wnt triggers the activation of G proteins to activate the Fz-mediated phospholipase, and subsequently trigger Ca^2+^ release and activate Ca^2+^-dependent effectors (Niehrs, 2012). In the spinal cord, Wnt1 and Wnt5a are expressed in a gradient along the anterior-posterior axis to regulate CST axons that extend through the spinal cord. Data on *Ryk* ^*−/−*^ mutants indicate that receptor-like tyrosine kinase (Ryk) functions as a Wnts receptor in the CST, and pharmacological *in vitro* experiments reveal that Ca^2+^ mediates the repulsive effect between Wnts and Ryk ^T^ (Liu et al., 2005; Ian et al., 2012).

Recently, an interaction between exosomes and Wnt/PCP signaling in cancer metastasis has been described. The activation of exosomes that are released by fibroblasts stimulate the protrusive activity and motility of breast cancer cells via Wnt/PCP signaling (Luga et al., 2012). Furthermore, exosomes mediate non-directional cell migration primarily in an actin-dependent, centrosome-independent manner (Luo et al., 2019). The Wnt/Ca^2+^ pathway induces exosome secretion in melanoma cells (Ekstrom et al., 2014). Moreover, in melanoma cells, Wnt5a stimulates exosomes to release their cargo, including immunomodulatory and pro-angiogenic proteins such as vascular endothelial growth factor and matrix metalloproteinase-2. This induction is blocked by the Ca^2+^ chelator BAPTA, inhibited by a dominant negative version of the small Rho-GTPase Cdc42, and is accompanied by cytoskeletal reorganization. Co-culture experiments demonstrate that blocking Wnt5a expression leads to endothelial cell morphological defects, whereas expressing Wnt5a in endothelial cells induces exosomes release from melanoma cells. These data suggest a role for the interaction between the Wnt/Ca^2+^ pathway and exosomes in immunosuppressive and angiogenic functions.

In the nervous system, active Wnt proteins are released by exosomes at the neuromuscular junction in *Drosophila* (Gross et al., 2012). Hippocampal interneuron-derived exosomes release endogenous proline-rich 7 (PRR7) which is the antagonist of Wnt signaling (Lee et al., 2018). Exosomal PRR7 is a transmembrane protein that can be taken up by neighboring neurons to eliminate excitatory synapses. PRR7 can block the exosomal secretion of Wnts and activation of glycogen synthase kinase-3 β by promoting proteasomal degradation of postsynaptic density proteins. Conversely, exosomes mediate Wnt signaling to contribute to axonal regeneration in central nervous system (CNS) injury (Tassew et al., 2017). Fibroblast-derived (FD) exosomes rescue neurite outgrowth defects when they are applied to cultured cortical neurons with inhibitory myelin substrate. This rescue ability is driven by the interaction between exosomes and Wnt10b, as FD exosomes promote the recruitment of Wnt10b towards lipid rafts which induces a Wnt10b autocrine signaling pathway that activates the mammalian target of rapamycin in neurons. Hence, applying FD exosomes to the eye promotes optic nerve regeneration after injury (Tassew et al., 2017). A recent bioinformatic analysis demonstrates that exosomes released by epiblast-derived stem cells carrying miRNA cargo that mediate Wnt signaling are significantly enriched during dopaminergic neuron differentiation (Jin et al., 2020). These studies highlight a key role for the exosome-Wnt interaction in both the developing and adult nervous system, including neurogenesis, axon outgrowth, and neuron differentiation, providing a new therapeutic target for CNS disorders. However, our understanding of exosomal Wnt in axon guidance remains limited.

Similar to long-range regulation by ephrins, non-canonical Wnt pathways may also act as long-range cues in guiding long-distance axonal pathfinding. In dopaminergic projections from the brainstem to midbrain targets, axons are extended along a gradient pattern of Wnt expression distributed along the existing tissue. This process may promote non-canonical Wnt signaling via neighboring neuron- or astrocyte-derived exosomes. Conversely, exosomal non-canonical cue interactions may indirectly mediate axonal pathfinding via crosstalk between guidance cues. For instance, Shh regulates the Wnt expression level to generate a Wnt expression gradient that regulates post-crossing commissural axons by inducing the expression of the Wnt antagonist Sfrp1 (Domanitskaya et al., 2010). As described previously, Wnt signaling mediates exosome release, which may activate exosomal guidance cues to regulate axonal pathfinding, suggesting a new model of exosome-dependent guidance cue crosstalk.

## 3 Role of exosomes in axonal outgrowth and regeneration

Nerve injuries, including traumatic brain, spinal cord, and peripheral injury, involve damage to nerve tissue that leads to communication defects within the brain or between the brain and other organs. Regenerating injured axons from the CNS injury and rebuilding functional circuits that connect to their original targets are difficult, and thus injury often results in permanent disability. However, peripheral nerves perform dramatic regeneration after injury leading to recovery of the function of sensory and motor innervations. Diverse surgical procedures have attempted to repair nerve injuries; however, several disadvantages remain due to donor tissue absence, functional nerve damage, and the risk of neuroma generation. Thus, a new therapeutic target for promoting or improving axonal outgrowth and regeneration is required.

Recently, exosomes have been widely studied and used as a popular therapeutic target. Exosomes can be used as an alternative non-cell-based therapy for nerve regeneration to reduce the risks of dysfunction and transformation of transplanted stem cells (Thirabanjasak et al., 2010). To achieve their contribution in nerve regeneration, exosomes act as vehicles for remyelinating and regenerative factors or miRNAs that mediate nerve regenerating events. Retinoic acid (RA), a well-known axon guidance molecule, plays an essential role in axon/neurite outgrowth and guidance during development and adulthood (Dmetrichuk et al., 2006; Farrar et al., 2009). The RA signaling pathway regulates remyelination and axon/neurite outgrowth after spinal cord injury, and, interestingly, exosomes mediate the neural-glial crosstalk that contributes to this process (Goncalves et al., 2018). Neuronal RA receptor beta (RARβ) activation is required for the RA signaling pathway in neural injury recovery and leads to the upregulation of endogenous RA synthesis and release of RA in exosomes as either cargo or anchored to the membrane to serve as a positive cue for axon growth, suggesting the importance of exosomes in nerve regeneration as guidance molecule carriers. As exosomes may carry miRNAs as cargo, numerous studies are focused on the molecular mechanism of exosomes in nerve regeneration on the gene level. Traumatic brain injury (TBI), the primary cause of acquired permanent neuronal disability worldwide, induces axonal transection and synapse dysfunction which results in neurological function deficits (Tsitsopoulos et al., 2017). Therefore, major therapeutic strategies for TBI focus on axonal regeneration and synapse recovery.

**FIGURE 2:**
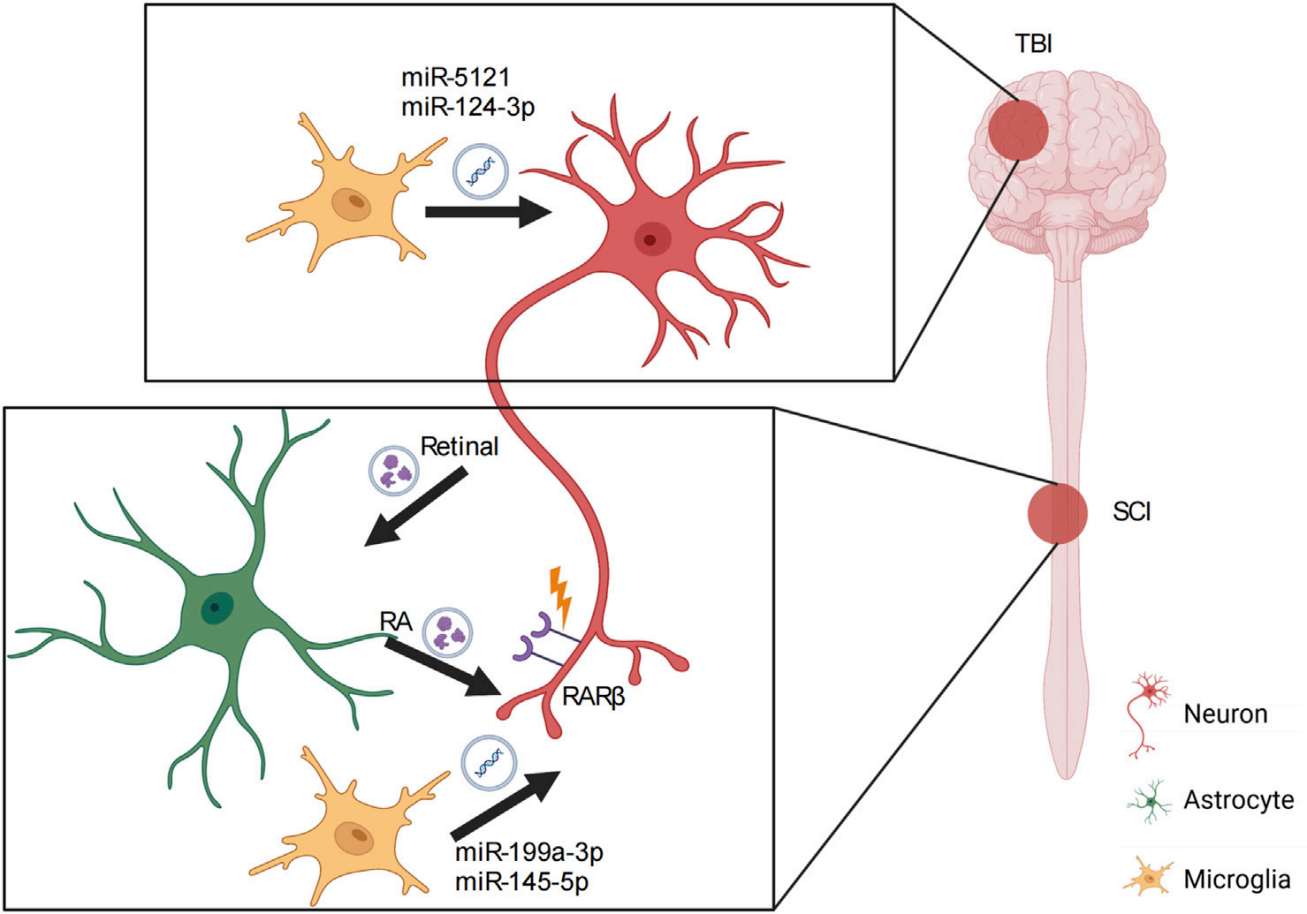
Role of exosomes in nervous system injury. In traumatic brain injury (TBI), microglial exosomes transfer miRNAs to the target neuron to induce neurite outgrowth and synapse recovery. In spinal cord injury (SCI), exosomes act as vehicles that carry both miRNAs and other molecules to support the interaction between neurons and astrocytes or neurons and microglia, contributing to axon recovery and subsequent axon pathfinding. RA, retinoic acid; RARβ, retinoic acid receptor beta.

Several studies demonstrate a key role of microglia in axon outgrowth and synaptic plasticity via crosstalk with neighboring neurons (Arnoux and Audinat, 2015), which is regulated by microglia-derived exosomes. After TBI, miR-5121 carried by microglial exosomes is significantly decreased, which may suppress neurite outgrowth and synapse recovery (Zhao et al., 2021). Overexpression of miR-5121 in a TBI model partly rescues the axonal and synaptic defects both *in vitro* and *in vivo*. Moreover, motor coordination in mice treated with exosomes overexpressing miR-5121 is significantly improved after fluid percussion injury compared with that in untreated animals. The results of gene ontology and Kyoto Encyclopedia of Genes and Genomes (KEGG) pathway analysis revealed that repulsive guidance molecule A is the downstream direct target of miR-5121 through which miR-5121 promotes axonal outgrowth and synapse recovery. In the spinal cord, interestingly, exosomes often promote neurite outgrowth by reducing inflammation at the lesion site, creating a favorable environment for neurite outgrowth (Wang et al., 2021). Exosomes carrying miR-199a-3p/145-5p contribute to SCI (Wang et al., 2021). miR-199a-3p/145-5p is highly expressed in exosomes that are derived from by-products of human umbilical cord mesenchymal stem cells and modulates the nerve growth factor/tropomyosin receptor kinase A (TrkA) pathway to regulate neuronal differentiation. Moreover, miR-199a-3p/145-5p increases TrkA expression at the lesion site to alleviate damage to the lesion site and facilitate locomotor function *in vivo*. Huang et al. (2018) highlighted the role of exosomal miR-124-3p in inhibiting neuronal inflammation and promoting neurite outgrowth. Furthermore, microglial exosomal miR-124-3p is significantly upregulated following TBI. miR-124-3p promotes anti-inflammatory M2 polarization in microglia to inhibit inflammation in scratch-injured neurons. Lastly, treatment with exosomal miR-124-3p rescues the decline in neurite number and length in a scratch injury model, accompanied by additional decreases in RhoA, amyloid precursor protein (APP), and Tau. These findings indicate a key role for exosomes in regulating axonal outgrowth and regeneration via their cargo of guidance cues or miRNAs in a direct or indirect manner (Figure 2).

## 4 Role of exosomes in neurodevelopmental disorders

Exosomes are thought to be expressed by all cell types in the nervous system to modulate crosstalk between them. During nervous system development, exosomes play key roles in neurogenesis, synaptogenesis, and circuit assembly to regulate neural circuit development (Sharma et al., 2019). During neural circuit development, defects in firing assembly and synapse formation result in numerous neurodevelopmental disorders (Gilman et al., 2012; Antonell et al., 2013; Iossifov et al., 2014; Parenti et al., 2020). This section summarizes the role of exosomes in some of these disorders and discusses the utility of exosomes as a cell-free therapy tool.

### 4.1 Autism spectrum disorders (ASD)

ASD is a well-known neurodevelopmental disorder that impacts emotion control, language learning, cognitive behavior, and social information perception abilities in children (Fodstad et al., 2009; Elsabbagh et al., 2011; Pierce et al., 2016). Increasing research has revealed that ASD is a largely heritable, multi-stage, and prenatal disorder (Courchesne et al., 2020). ASD is associated with abnormalities in multiple brain regions and other organs. Moreover, ASD is related to a combination of abnormal nerve development events, including neurogenesis, cell migration, axon outgrowth, spine development, and synaptogenesis in prenatal and early postnatal life (Courchesne et al., 2011; Kaushik and Zarbalis, 2016; Packer, 2016; Marchetto et al., 2017). Studies on inducible pluripotent stem cells (iPSC) derived from individuals with ASD have demonstrated disruptions in neural activity, cell proliferation, and synaptogenesis in children with ASD. An iPSC study has revealed the misregulation of genes involved in neuronal differentiation, axon guidance, cell migration, and regional patterning, however, there are barely no genes have a definite relation with ASD and this disease is eventuated by hundreds of genses in neurodevelopment and synaptic proteins (Iakoucheva et al., 2019; DeRosa et al., 2018). Consistently, a GWAS analysis validated ASD risk genes and determined that most of these genes were expressed in ASD-implicated brain regions, including the neocortex, cerebellum, amygdala, hippocampus, and striatum (Krishnan et al., 2016).

Currently, exosomes have been implicated as potential biomarkers to diagnose ASD in early childhood, as no specific biomarker of ASD exists and reliable biomarkers found in exosome cargos, such as abundant miRNAs, may be easily obtained from different types of body fluid (Nouri et al., 2024; Chen et al., 2023; Liu et al., 2024). Moreover, exosomes play key roles in regulating immune system imbalances in patients with ASD, and thus may be used as a drug delivery system to reverse immunological defects in patients with ASD (Chen et al., 2016; Li et al., 2019; Xian et al., 2019). However, the role of exosomes in regulating neural circuit defects in ASD is unknown. Several studies show that diverse resources of exosomes play key roles in axon outgrowth, cell migration, neurogenesis, and synaptogenesis during development. Furthermore, Zhdanova et al. (2021) intranasally administered exosomes derived from multipotent mesenchymal stromal cells in patients with Alzheimer’s Disease (AD) and detected the labeled exosomes in the neocortex and hippocampus. These findings demonstrate the potential therapeutic utility of exosomes in treating ASD at an early postnatal stage.

### 4.2 Intellectual disability and down syndrome (DS)

Intellectual disability is commonly defined as below-average intellectual functioning before 18 years of age, IQ score below 70, and defects in communication, self-care, and social skills. Several factors are linked to the development of intellectual disability, including genetic disorders, trauma, and prenatal events, including maternal infection and alcohol exposure. However, in almost half of these cases, the pathological mechanisms leading to intellectual disability are unknown (Daily et al., 2000; Schroeder, 2000). DS is the most prevalent intellectual disability that is also associated with phenotypical defects, including congenital heart disease and other developmental abnormalities. DS is a non-lethal genetic developmental disorder with a high incidence rate and a global morbidity of 1/800. It is described as a cognitive defect with development of age-dependent neuropathology, such as Alzheimer’s disease (AD). Before dementia occurs, biomarkers, including amyloid-β (Aβ) peptides, p-Tau protein, and interleukin-6, can be detected in the CSF of young patients with DS (Counts et al., 2017). However, collecting CSF poses risks of invasiveness and post-lumbar puncture headaches, particularly in young patients (Hamlett et al., 2017).

In the last 5 years, research on exosomal secretion in DS using *post mortem* brain tissue has demonstrated an approximate 40% increase in exosomes detected in DS samples compared with that in control tissue (Gauthier et al., 2017). Hamlett et al. (2018) showed a similar increase in neuron-derived exosomes in blood samples from patients with DS and determined that the Aβ1-42, p-T181-Tau, and p-S396-Tau cargos in DS neuron-derived exosomes were significantly increased compared with that in the control. These findings suggest that exosomes may be used as potential biomarkers to diagnose the early dementia phenotype of DS while avoiding the risks associated with CSF collection. Furthermore, these data reveal a new source of APP-related protein transport by neuron-derived exosomes in the brain and potentially non-brain regions in the body. Lastly, peripheral metabolism of Aβ is associated with the risk of AD, and Aβ synthesis in the liver may result in a neurodegenerative phenotype (Lam et al., 2021). Together, a new model of bidirectional brain-body transport of Aβ loaded in exosomes may be hypothesized.

## 5 Conclusion

Numerous studies have highlighted the relevant roles of exosomes in nervous system development and neurodegenerative diseases. As a potential biomarker, exosomes are easily collected in body fluids such as blood and saliva, providing an advantage over other collection methods. Furthermore, exosomal miRNAs are more resistant to degradation and easier to isolate than free miRNAs in body fluids. As exosomes are small, present low immunogenicity, lack the ability to transform cells, and are highly therapeutic, they can be used as disease diagnosis markers, drug delivery vehicles, and therapeutic agents for various diseases involving CNS pathology and injury.

Various miRNAs related to axon guidance and neuroplasticity are loaded in exosomes derived from many cell types. In addition to these miRNAs, exosomes carry or bind to canonical guidance cue ligands or receptors resulting in the regulation of long-range axonal pathfinding. Moreover, exosome cargo miRNAs mediate non-canonical guidance cues, such as Wnt and Shh, to activate neurite/axon outgrowth and regeneration. Most axon guidance cues, non-conventional cues, and related miRNAs are carried by, expressed, or interact with exosomes. However, studies on the role of exosomes in neural circuit development is focused on cell-cell communication, nerve regeneration, and synapse formation. Axon guidance plays a key role in neural circuit development to regulate the correct targeting of 80 billion neurons in the brain. Clinically, axon guidance is also implicated in numerous neurodevelopmental and neurodegenerative disorders, including ASD, DS, AD, and Parkinson’s disease. Axon guidance is a key step in ensuring that the regenerated axons project to their original targets during nerve injury recovery. In the latest studies, researchers seem to prefer using specifically derived exosomes as a “pathway candidate pool” to better understand the contents of functional exosomes, which have been shown to have positive therapeutic effects on certain disorders (Tang et al., 2024; Wu et al., 2024; Coy-Dibley et al., 2024; Liu et al., 2024). This trend illustrates a new approach to characterizing exosomes from “inside”, focusing not on generalities, but on their specific functions.
